# The Constant Threat of Zoonotic and Vector-Borne Emerging Tropical Diseases: Living on the Edge

**DOI:** 10.3389/fitd.2021.676905

**Published:** 2021-05-04

**Authors:** Alfonso J. Rodriguez-Morales, Alberto E. Paniz-Mondolfi, Álvaro A. Faccini-Martínez, Andrés F. Henao-Martínez, Julian Ruiz-Saenz, Marlen Martinez-Gutierrez, Lucia E. Alvarado-Arnez, Jorge E. Gomez-Marin, Ruben Bueno-Marí, Yenddy Carrero, Wilmer E. Villamil-Gomez, D. Katterine Bonilla-Aldana, Ubydul Haque, Juan D. Ramirez, Juan-Carlos Navarro, Susana Lloveras, Kovy Arteaga-Livias, Cristina Casalone, Jorge L. Maguiña, Angel A. Escobedo, Marylin Hidalgo, Antonio C. Bandeira, Salim Mattar, Jaime A. Cardona-Ospina, Jose A. Suárez

**Affiliations:** 1 Grupo de Investigación Biomedicina, Faculty of Medicine, Fundacion Universitaria Autonoma de las Americas, Pereira, Colombia; 2 Emerging Infectious Diseases and Tropical Medicine Research Group, Instituto para la Investigación en Ciencias Biomédicas - Sci-Help, Pereira, Colombia; 3 Coordinación Nacional de Investigación, Universidad Privada Franz Tamayo (UNIFRANZ), Cochabamba, Bolivia; 4 Master Program on Clinical Epidemiology and Biostatistics, Universidad Científica del Sur, Lima, Peru; 5 Department of Pathology, Molecular and Cell-Based Medicine, Laboratory of Microbiology, Icahn School of Medicine at Mount Sinai, New York, NY, United States; 6 Instituto de Investigaciones Biomédicas IDB/Incubadora Venezolana de la Ciencia, Barquisimeto, Venezuela; 7 Department of Pathology, University of Texas Medical Branch, Galveston, TX, United States; 8 Department of Medicine, Division of Infectious Diseases, School of Medicine, University of Colorado Denver, Aurora, CO, United States; 9 Grupo de Investigación en Ciencias Animales - GRICA, Facultad de Medicina Veterinaria y Zootecnia, Universidad Cooperativa de Colombia, Bucaramanga, Colombia; 10 Infettare, Facultad de Medicina, Universidad Cooperativa de Colombia, Medellín, Colombia; 11 Grupo de Estudio en Parasitologia Molecular (GEPAMOL) Group, Facultad de Ciencias de la Salud, Universidad del Quindío, Armenia, Colombia; 12 Departamento de Investigación y Desarrollo (I+D), Laboratorios Lokímica, Paterna, Spain; 13 Área de Parasitología, Departamento de Farmacia y Tecnología Farmaceútica y Parasitología, Universidad de Valencia, Burjasot, Spain; 14 Facultad de Ciencias de la Salud, Carrera de Medicina, Universidad Técnica de Ambato, Ambato, Ecuador; 15 Infectious Diseases and Infection Control Research Group, Hospital Universitario de Sincelejo, Sincelejo, Colombia; 16 Programa Del Doctorado de Medicina Tropical, SUE Caribe, Universidad Del Atlántico, Barranquilla, Colombia; 17 Semillero de Investigación en Zoonosis (SIZOO), Grupo de Investigación BIOECOS, Fundacion Universitaria Autonoma de las Americas, Pereira, Colombia; 18 Department of Biostatistics and Epidemiology, University of North Texas Health Science Center, Fort Worth, TX, United States; 19 Centro de Investigaciones en Microbiología y Biotecnología-UR (CIMBIUR), Facultad de Ciencias Naturales, Universidad del Rosario, Bogotá, Colombia; 20 Research Group of Emerging Diseases, Ecoepidemiology and Biodiversity, Health Sciences Faculty, Universidad Internacional SEK, Quito, Ecuador; 21 Sección Zoopatología Médica, Hospital de Infecciosas FJ Muñiz, Buenos Aires, Argentina; 22 Faculty of Medicine, Universidad Nacional Hermilio Valdizán, Huánuco, Peru; 23 Istituto Zooprofilattico del Piemonte, Torino, Italy; 24 Department of Epidemiology, Institute of Gastroenterology, Havana, Cuba; 25 Infectious Diseases Group, Facultad de Ciencias, Pontificia Universidad Javeriana, Bogotá, Colombia; 26 Faculdade de Medicina, UniFTC, Salvador, Brazil; 27 Instituto de Investigaciones Biologicas del Tropico, Universidad de Cordoba, Monteria, Colombia; 28 Investigador SNI Senacyt Panamá, Instituto Conmemorativo Gorgas de Estudios de la Salud (ICGES), Panama, Panama

**Keywords:** vector-borne diseases, zoonotic diseases, emerging, SARS-CoV-2/COVID-2, tropical diseases, challenges

Emerging diseases have significantly impacted the last few decades (1–10). The emergence and re-emergence of vector-borne and zoonotic diseases in Africa, Asia, and Latin America have reshaped the epidemiological landscape of these continents. The impact of these diseases and the establishment of local transmission in traditionally non-endemic areas, due to migration and travel, have been revealed over the last years. Diseases such as Chikungunya (11–16), Zika (17–24), Yellow Fever (25–28), Dengue (29–33), Oropouche, Madre de Dios virus, Iquitos virus (34, 35), Mayaro Fever (36, 37), Ebola (38–42), Nipah virus, arenaviruses such as Lassa (43), Machupo (44, 45), Chapare (45, 46), Junin (47), zoonotic Malaria (48), Severe Fever with Thrombocytopenia Syndrome (49), Plague (50), Crimean-Congo Hemorrhagic Fever, Acute Orally Transmitted Chagas Disease (51–54), Visceral and Diffuse Cutaneous Leishmaniasis (55, 56), Toxoplasmosis (57–59), Tick-Borne Diseases (60, 61), Rift Valley Fever, Tuberculosis (62), Leprosy (63–67), Avian Influenza (68–70), Orthohantavirus (71–75), and Toxocariasis (76, 77) have posed a significant impact to human health. Furthermore, zoonotic epidemics and pandemic coronaviruses, such as the Severe Acute Respiratory Syndrome (SARS), the Middle East Respiratory Syndrome (MERS) (78–82), and the ongoing SARS-CoV-2/COVID-19 (83, 84) pandemic, have caused a profound economical and social disruption threatening to overwhelm public health systems globally (85) (**Table 1**). Most of these pathogens can even cocirculate and coinfect a significant proportion of inhabitants within the same territories (11, 87–94). For example, in arboviral diseases, the occurrence of coinfections has been widely reported –such as Dengue with Chikungunya and/or with Zika virus– and affects diverse populations, including pregnant women and immunocompromised patients (94–97). This may obscure clinical suspicion, as signs and symptoms for many of these pathogens may overlap. In endemic areas, this becomes a particularly pressing issue that must be taken into account in order to ensure accurate diagnosis and provide appropriate management. The ChikDenMaZika syndrome has been previously adopted as a mnemonic device to include Chikungunya, Dengue, Mayaro, and Zika in the broad differential of acute febrile illnesses due to arboviral agents (95). More recently, emerging coinfections, including bacterial and parasitic diseases, such as tuberculosis and Chagas disease, have also been reported (98).

**Table 1:** Lessons learned from the COVID-19 pandemic in Latin America.

• Avoid fragmentation and segmentation of the health system
• Enhance data integration between sectors
• Improve transfer of inputs and deployment of personnel
• Better linking of health and safety authorities
• Build up a strong capacity for molecular (RT‐PCR) testing
• Validate rapid tests for complementary diagnosis
• Improve primary care interventions before admission to ICU
• Improve ICU capacities including facilities, equipment, and personnel
• Better management and monitoring of non‐COVID patients
• Promote education of human resources, including health professionals
• Improve health personnel’s working conditions (salaries, PPE, among others)
• Enhance medical training during the pandemic
• Warrant medicinal oxygen supplement
• Monitor transparency in health authorities’ decision‐making documents
• Use of medications with evidence and develop evidence-based guidelines
• Provide appropiate information about public health policy and decision‐making processes
• Develop more capacities in biotechnology (for development of tests, treatments, and vaccines)

Modified from Herrera-Añazco et al. (86).

Current times call for more comprehensive ecoepidemiological and bioecosocial approaches (20, 99). Scarce funding and the lack of research (39, 43, 61, 81) in tropical medicine are entirely unacceptable. Human immunodeficiency virus (HIV)/acquired immune deficiency syndrome (AIDS), tuberculosis (TB), and malaria combined receive approximately 70% of neglected diseases funding. As mentioned here, emerging tropical diseases, such as those mentioned here, are worldwide in scope, and many have significant regional implications. Therefore, a different funding paradigm that improves their situation is needed (100). The world is no longer a place with distant countries and shielded territories. Instead, ever increasing interconnectivity has turned it into a “small” global village, where the health status of underprivileged areas may undermine not only their lives and development but extend to the wealthiest. The Ebola crisis in 2014 highlighted how high-consequence emerging diseases could spill over to Europe and North America (38, 40). The ongoing 2020-2021 pandemic of COVID-19, which has reached as far as Antarctica, affecting almost all countries worldwide, is another clear example (8, 29, 84, 101–112). As was expected, coinfections between tropical pathogens and COVID-19 are also now increasingly being reported, especially with dengue (30). Dengue affects over 100 countries worldwide and puts about 2.5-3.9 billion people at risk of infection (113, 114). Within the next century, nearly a billion people are at risk of exposure to virus transmission by both main *Aedes* spp., *Ae. aegypti*, and *Ae. albopictus* (also Chikungunya and Zika) in the worst-case scenario (115). The recent first detection of *Ae. vittatus* in the Dominican Republic and the Americas generated concern in the region, requiring enhanced surveillance to understand the range and public health risks of this potential invasive mosquito species, deserving more studies (116). Most of these emerging tropical diseases are vector-borne, zoonotically transmitted, or environmentally spread through direct contact, food or water ingestion, as well as a consequence of environmental alterations (including the effects of climate change) (117–125), becoming significant sources of mortality and morbidity worldwide (2).

The impact of these diseases extends well beyond the acute constellation of symptoms, leading in a considerable proportion of patients to chronic sequelae and complications, which can be long lasting and severely incapacitating, as is the case with Chikungunya (15, 126–132), Zika (17, 133–135), Ebola, Chagas disease (52), and even for COVID-19 (136–139).

Many tools have been deployed to counteract emerging infectious diseases. Amongst these are active surveillance (some supported by artificial intelligence) (140–142), leading to the rapid identification of novel pathogens by genome sequencing and phylogenetic tracing studies (36, 105, 107, 143–146) based on computing methods to predict possible interspecies barriers spillover between humans and animals (147). Coupling biotechnological approaches with social sciences—the holistic understanding of humans and their interactions in the disease ecosystems—is also a critical element needed when studying emerging infectious diseases (148, 149).

One of the most significant challenges when studying tropical infectious diseases relies on their complexity and heterogeneity, which usually requires a deep understanding not only of the disease itself but its overall context. In order to better approach these diseases one must keep a broader vision of designing proposed interventions, including multilevel ecoepidemiological studies ranging from molecular and omics to satellite epidemiology (use of data and images derived from geospatial technologies, e.g., satellites, for the study of the occurrence and distribution of health-related events in specified populations, and the application of this knowledge to control the health problems) of pathogens, vectors, hosts, abiotic variables, and other socio-environmental factors (125, 150, 151). While more research is required to fill in the numerous gaps in knowledge for many of these diseases, particular attention should be placed in designing strategies to develop methods to forecast these diseases not only in vulnerable and underserved populations from low-income countries but also in those poverty pockets located in high-income countries. A whole chapter to be considered in emerging tropical diseases is vaccines development. Innovative global partnership between public, private, philanthropic, and civil society organisations, such as the Coalition for Epidemic Preparedness Innovations (CEPI), launched in 2017, are important to develop vaccines to stop future epidemics. To accelerate the development of vaccines against emerging infectious diseases and enable equitable access to these vaccines for people during outbreaks is crucial. Nevertheless, more funding to understand biology, pathogenesis, epidemiology, prevention, and treatment of emerging tropical diseases are urgently needed and expected (152–154).

Tropical Medicine is no more a clinical specialty of “exotic diseases,” as it was conceived at its beginnings, and is no more about “diseases for those entering the jungle.” One dramatic change is the urban installation of diseases that before were observed only after sylvatic or primary forest exposure. The increase of urban outbreaks of Chagas disease in South America is now a horrific reality in Brazil (155–157), Venezuela (158), and Colombia (159, 160), and it is also a new reality for visceral leishmaniasis (161–164). The integrated work of public health experts, veterinarians, entomologists, and parasitologists is an urgent need to face these new challenges and transformations of tropical diseases. Tropical diseases also include non-infectious diseases, such as animal bites and stings (e.g. myiasis and tungiasis) (165, 166). Snake bites, scorpion stings, and spider bites, account for a significant amount of the morbidity and mortality in tropical countries in these changing scenarios, including ecotourism, rural migration, and other related factors (167–170).

There is no doubt that “many things are wrong in the world today”, as the legendary American rock n’roll band Aerosmith has been singing since the 90s. We are “living on the edge”, the edge of neglect and of a surge of many emerging infectious diseases with no hope for resolution in the foreseeable future. Furthermore, “it sure ain’t no surprise” that poverty, inequality, climate change, deforestation, migration, urbanization, wildlife trade, among many other factors, have all contributed to the emergence of novel tropical diseases and the resurgence of other endemic diseases (171). There is no spare place for the arrival of emerging pathogens, and over time pathogens tend to adapt to new environments leading to unforeseen consequences. The next epidemic, the next pandemic, is just around the corner (68). In response to this latent threat, we need to gather real-time information and build collaborative networks aimed to enhance surveillance activities in order to develop high-priority medical countermeasures to prevent and control emerging tropical diseases. Research in Zoonotic and Vector-Borne Emerging Tropical Diseases remains the most critical aspect and the foundation to determine the drivers of emerging and re-emerging infectious diseases.

With that vision, our new Section *Emerging Tropical Diseases* in the journal Frontiers in Tropical Diseases offers to contribute to the scientific advancement and fill in the many knowledge gaps based on a multi and transdisciplinary approach. Our team of Associate Editors is comprised of a diverse group of experts from different countries, diverse backgrounds, and varied interrelated expertises in a wide range of conditions within the tropical diseases spectrum of diseases, following the One Health approach vision (8, 172).

Grand challenges exist in the fight against the threat of emerging tropical diseases. In the laboratory, our daily work, in the hospitals, in the field, in the community, and in many other places, our shared goal is to understand the drivers of emergence and address their root-causes. We are working collaboratively in social networks to reduce the impact of emerging tropical diseases. Let’s work on this together! We value your work and welcome your submissions to this new section of *Frontiers in Tropical Diseases*.

## Author Contributions

All authors contributed to manuscript conception and design, literature review, manuscript preparation, and critical review. All authors contributed to the article and approved the submitted version.

## Conflict of Interest

RB-M was employed by Laboratorios Lokímica, Spain.

The remaining authors declare that the research was conducted in the absence of any commercial or financial relationships that could be construed as a potential conflict of interest.
